# Osteophytes mediate the associations between cartilage morphology and changes in knee symptoms in patients with knee osteoarthritis

**DOI:** 10.1186/s13075-022-02905-8

**Published:** 2022-09-08

**Authors:** Tianxiang Fan, Shibo Chen, Muhui Zeng, Jia Li, Xiaoshuai Wang, Guangfeng Ruan, Peihua Cao, Yan Zhang, Tianyu Chen, Qianhua Ou, Qianyi Wang, Anita E. Wluka, Flavia Cicuttini, Changhai Ding, Zhaohua Zhu

**Affiliations:** 1 Clinical Research Centre, Zhujiang Hospital, Southern Medical University, Guangzhou, Guangdong China; 2 Division of Orthopaedic Surgery, Department of Orthopaedics, Nanfang Hospital, Southern Medical University, Guangzhou, Guangdong China; 3 Department of Epidemiology and Preventive Medicine, Monash University, Melbourne, Victoria Australia; 4 Menzies Institute for Medical Research, University of Tasmania, Hobart, Tasmania Australia; 5 Department of Orthopaedics, Zhujiang Hospital, Southern Medical University, Guangzhou, Guangdong China

**Keywords:** Knee osteoarthritis, Cartilage, Knee pain, Osteophytes, Magnetic resonance imaging

## Abstract

**Aims:**

To investigate whether the associations between cartilage defects and cartilage volumes with changes in knee symptoms were mediated by osteophytes.

**Methods:**

Data from the Vitamin D Effects on Osteoarthritis (VIDEO) study were analyzed as a cohort. The Western Ontario and McMaster Universities Osteoarthritis Index was used to assess knee symptoms at baseline and follow-up. Osteophytes, cartilage defects, and cartilage volumes were measured using magnetic resonance imaging at baseline. Associations between cartilage morphology and changes in knee symptoms were assessed using linear regression models, and mediation analysis was used to test whether these associations were mediated by osteophytes.

**Results:**

A total of 334 participants (aged 50 to 79 years) with symptomatic knee osteoarthritis were included in the analysis. Cartilage defects were significantly associated with change in total knee pain, change in weight-bearing pain, and change in non-weight-bearing pain after adjustment for age, sex, body mass index, and intervention. Cartilage volume was significantly associated with change in weight-bearing pain and change in physical dysfunction after adjustment. Lateral tibiofemoral and patellar osteophyte mediated the associations of cartilage defects with change in total knee pain (49–55%) and change in weight-bearing pain (61–62%) and the association of cartilage volume with change in weight-bearing pain (27–30%) and dysfunction (24–25%). Both cartilage defects and cartilage volume had no direct effects on change in knee symptoms.

**Conclusions:**

The significant associations between cartilage morphology and changes in knee symptoms were indirect and were partly mediated by osteophytes.

**Supplementary Information:**

The online version contains supplementary material available at 10.1186/s13075-022-02905-8.

## Introduction

Osteoarthritis (OA) affects more than 40% of individuals over the age of 70 years [1] and is the leading cause of pain and loss of physical function [2]. There are no authorized treatments that have been proven to slow the progression of OA. Articular cartilage is a central hallmark in OA and has been long viewed as a major target tissue for OA treatment. Despite the lack of nociceptive fiber, cartilage morphology has been found to be associated with knee pain and dysfunction. A cross-sectional analysis from the Osteoarthritis Initiative (OAI) study reported that cartilage lesions were more frequent in subjects with knee pain compared to those without knee pain [3]. Wluka et al. reported that there was a significant association between tibial cartilage volume and symptoms at baseline and a weak but significant association between decreased articular cartilage volume and worsening symptoms of OA over 2 years [4]. Another study from OAI reported that a reduction in central medial femorotibial cartilage thickness was associated with pain progression [5]. However, the underlying mechanism for the relationship between loss of articular cartilage and deterioration of symptoms is still unclear.

Mediation analysis is a method to estimate the extent to which an intervention or exposure may affect an outcome through a potential causal mechanism [6]. The value of mediation analyses has been recognized by national funding organizations such as the US National Institutes of Health and the UK National Institute for Health Research [7, 8]. A recent high-quality study reported that cartilage thickness loss was associated with worsening knee pain, while only a small proportion of the association was mediated by worsening synovitis but not bone marrow lesions (BMLs) [9]. This study raises important concerns about whether the remaining association between cartilage morphology and knee symptoms was also mediated by other structural abnormalities. Cartilage defects and cartilage volumes are both well-recognized assessments of cartilage morphologies, but they emphasize the different aspects of cartilage. To comprehensively represent cartilage status, both of them were included in the analysis.

The osteophyte has also been considered to be an important source of knee pain. Sensory and sympathetic innervations have been observed in the bone marrow cavity of osteophytes [10]. Perivascular substance P-immunoreactive nerve fibers have also been localized to the bases of osteophytes [11]. It is well established that the presence of osteophytes has positive associations with knee symptoms [12–14]. Endochondral ossification has been considered as the most important process in the formation of osteophytes [15]. The degradation of cartilage is also associated with osteophyte formation [16–18]. Our previous study reported that baseline cartilage defects could predict an increase in MRI-detected osteophytes over 2.6 years after adjustment for baseline osteophyte score, BMLs, and meniscal extrusion [19]. Hence, we hypothesize that the associations between cartilage morphology and knee symptoms can be mediated by osteophytes.

Our aims were therefore to investigate the association of cartilage defects and cartilage volume with a change in knee symptoms and to explore whether osteophytes mediate the relationship between them.

## Methods

### Participants

**Fig. 1:**
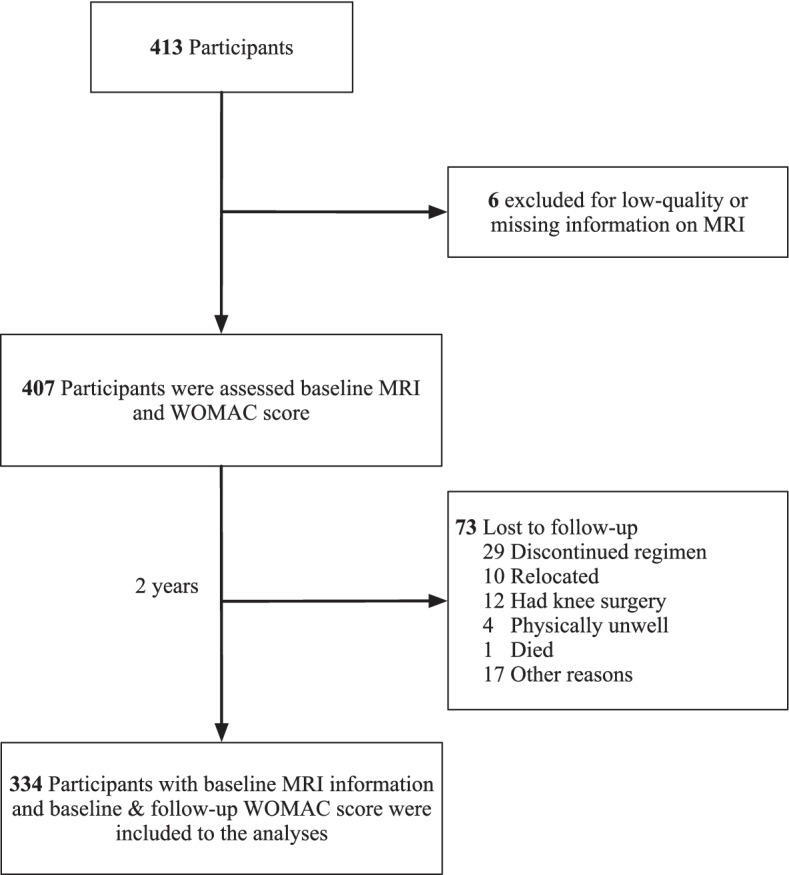
Flowchart for the study sample

This study used data from the Vitamin D Effects on Osteoarthritis (VIDEO) study, a randomized, double-blind, and placebo-controlled clinical trial that was originally designed to examine the effects of vitamin D supplementation on OA [20]. Informed written consent was obtained from all participants. The detailed study protocol including the inclusion and exclusion criteria has been published [20]. A total of 413 individuals aged from 50 to 79 (mean age 63.2 years) with symptomatic knee OA and vitamin D insufficiency (12.5 to 60 nmol/L) were selected from Victoria and Tasmania, Australia, through advertising in the community and recommended by doctors. The VIDEO study was approved by the Tasmania Health and Human Medical Research Ethics Committee and Monash University Human Research Ethics Committee and obtained consent from each participant. Participants had baseline measures between June 2010 and December 2011 and had follow-up examinations 2 years later. The data was analyzed as a cohort in the current study, and the analyses included 334 (80.9%) out of the original 413 participants with MRI information and measured the Western Ontario McMaster Osteoarthritis Index (WOMAC) score (baseline and follow-up). Six participants were excluded due to low-quality or missing information on MRI, and 73 participants were excluded due to loss of follow-up for discontinued regimen, knee surgery, relocation, physically unwell, death, and some other reasons (Fig. 1). The knees that met the previously described inclusion and exclusion criteria were selected as study knees to assess the outcomes [21]. When both knees met the criteria, the study knee was defined as the one with worse pain assessed using the visual analog scale.

### Anthropometrics

Electronic scales (Heine S-7307, Heine, New Hampshire, USA) were used to measure the weight, accurate to 0.1 kg (without bulky clothing and shoes). Stadiometer (Leicester Height Measure, Invicta Plastics Ltd, Leicester, UK) was used to measure the height, accurate to 0.1 cm. Body mass index (BMI) was calculated (BMI = weight (kg)/height^2^ (m^2^)).

### Magnetic resonance imaging

A commercial transmit-receive extremity coil 1.5-T whole-body magnetic resonance unit (Picker, Cleveland, OH, USA) was employed to image the knee structures at baseline and follow-up. The following image sequences were used: (1) a T1-weighted fat saturation spoiled gradient echo (GRE) sequences, flip angle 30°, repetition time 31 ms, echo time 6.71 ms, field of view 16 cm, 60 partitions, 512 × 512-pixel matrix, acquisition time 5 min 58 s, and 1 acquisition; sagittal images were obtained at a partition thickness of 1.5 mm without between-slice gap; (2) a T2-weighted/proton density-weighted fat saturation 3-dimensional fast spin echo, flip angle 90°, repetition time 3067 ms, echo time 112 ms, field of view 16 cm/15 partitions, and 228 × 256-pixel matrix; sagittal images were obtained at a partition thickness of 2 mm with a between-slices gap of 0.5–1.0 mm. Osirix (University of Geneva, Geneva, Switzerland) was applied to browse the image database on an independent computer as previously described [22, 23].

### MRI-detected osteophytes

MRI-detected osteophyte was measured in fat-saturated T1-weighted spoiled gradient echo (GRE) using a method which integrates the Whole-Organ Magnetic Resonance Imaging Score (WORMS) and the Knee Osteoarthritis Scoring System (KOSS) [24, 25] by TF who had over 2 years of experiences as an orthopedist. By definition, osteophyte was focal bony excrescence extending from a cortical surface which was detected on axial, sagittal, or coronal images. The line distance from the base to the tip was measured as the length of osteophytes [26]. Osteophyte was categorized into four grades based on their length: grade 0, absent; grade 1, < 3 mm; grade 2, 3–5 mm; and grade 3, > 5 mm [25]. We measured osteophyte in 14 subregions: femoral condyles and tibial plateaus were divided into anterior (a), central (c), and posterior (p) margins on sagittal, medial (M), and lateral (L) margins on coronal images. The patella was divided into medial (M) and lateral (L) margins. The sum score of each individual site in the relevant compartment was regarded as the osteophytes score in that compartment. Osteophyte scores in the lateral tibiofemoral (LTF) compartment, medial tibiofemoral (MTF) compartment, and patellar compartment were used as exposures for the analyses. The method was established by Zhu et al. and has been published elsewhere [12, 19, 27]. MRI-detected osteophytes were remeasured by ZZ and WH in 40 randomly selected participants, with a 4-week interval, to calculate the intra-observer and inter-observer reliabilities. Intra-observer reliability (expressed as intraclass correlation coefficients (ICCs)) was 0.94–0.97, and inter-observer reliability was 0.90–0.96 [19].

### Cartilage defects

Cartilage defects were graded on T2-weighted images at the medial tibial, medial femoral, lateral tibial, lateral femoral, trochlear groove, and patellar sites: grade 0, normal cartilage; grade 1, focal blistering and intracartilaginous low-signal intensity area with an intact surface and bottom; grade 2, irregularities on the surface or bottom and loss of thickness of less than 50%; grade 3, deep ulceration with loss of thickness of more than 50%; and grade 4, full-thickness chondral wear with exposure of subchondral bone [20]. The baseline total score was calculated as the sum of subregional scores and was used as an exposure. Intra-observer reliability expressed as an intraclass correlation coefficient ranged from 0.77 to 0.94 [21].

### Cartilage volume

The volumes of individual cartilage plates (medial tibial, lateral tibial, and patella) were isolated from the total volume by manually drawing disarticulation contours around the cartilage boundaries on a section-by-section basis. Sagittal images were obtained at a partition thickness of 1.5 mm and an in-plane resolution of 0.31 × 0.31 mm (512 × 512 pixels), then resampled by means of bilinear and cubic interpolation (area of 312 μm × 312 μm and multiplied by 1.5 mm thickness, continuous sections) for the final 3-dimensional rendering using OsiriX Lite imaging software (32-bit version 5.9, Pixmeo SARL). Particular cartilage volume was then determined by summing all the pertinent voxels within the resultant binary volume. The baseline total cartilage volume was the sum of medial tibial, lateral tibial, and patella cartilage volume and was used as an exposure. The coefficient of variation was 2.1% for the medial tibia, 2.2% for the lateral tibia, and 2.6% for the patella [28].

### Knee symptoms assessments

We measured the subscales of the WOMAC [29] including knee pain (5 items, 0–500), knee stiffness (2 items, 0–200), and knee dysfunction (17 items, 0–1700) at baseline and follow-up. Knee pain was categorized as weight-bearing pain (flat surface walking, going up/downstairs, and standing upright) and non-weight-bearing pain (in bed at night and sitting/lying). Total WOMAC pain score, total stiffness score, and total dysfunction score were the sum score of 5 pain items, 2 stiffness items, and 17 dysfunction items, respectively. The WOMAC change scores were calculated as the score at the follow-up visit minus the score at the baseline visit. WOMAC subscales (pain, stiffness, and physical function) were internally consistent with Cronbach’s coefficient alpha of 0.91, 0.81, and 0.84, respectively [30]. Intraclass correlation coefficients (ICCs) of WOMAC subscales (pain, stiffness, and physical function) were 0.86, 0.68, and 0.89, respectively [30].

### Statistical analysis

Multivariable linear regression was used to determine the associations between cartilage defects/cartilage volume and changes in WOMAC total pain, weight-bearing pain, non-weight-bearing pain, stiffness, and physical dysfunction, adjusted for age, sex, BMI, and intervention.

**Fig. 2:**
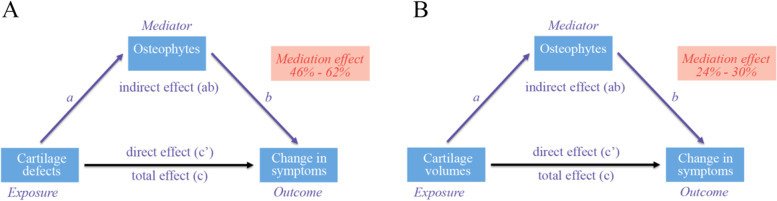
Model of the potential mediating effect of osteophytes on the relationship between cartilage morphology and changes in knee symptoms

The R package “Mediation” of the R studio software was used to explore how the total effect of cartilage morphologies on symptom changes to 2 years might be broken down into the “controlled direct effect” of cartilage morphology on pain and the “indirect effect” on a pathway through osteophytes (Fig. 2). Mediation analyses were estimated using bootstrapping (2000 replications) to recover the correct 95% confidence interval (CI) for direct and indirect effects. Mediation was estimated separately for LTF osteophyte, MTF osteophyte, and patellar osteophyte. The mediation percentage was then obtained by dividing the mediation effect by the total effect. It was considered a significant mediation effect if both the total effect and the mediating effect were statistically significant. The regression coefficient (*β*) of *P* value < 0.05 (two-tailed) or 95% CI did not include 0, indicating that there was a statistically significant association.

## Results

### Baseline characteristics of participants

The characteristics of the study sample are shown in Table 1. A total of 334 participants (46% female) with a mean age of 63.3 (SD = 7.2) and a BMI of 29.4 (SD = 4.9) were included in the analysis. Most baseline characteristics were comparable between participants who completed the study and those who did not, except that those who withdrew had more females and lower baseline cartilage volume (Additional file 1: Table S5). The baseline median score of osteophytes in the MTF compartment was 4.0, in the LTF compartment 4.0, and in the patellar compartment 2.0. The baseline median total cartilage defects score was 15.0, and the baseline median total cartilage volume was 5.7 cm^3^. The baseline median WOMAC pain score was 115.0 and decreased by 33.0 over 24 months. The baseline median weight-bearing pain score was 79.5 and decreased by 21 over 24 months. The baseline median non-weight-bearing pain score was 32.0 and decreased by 8.0 over 24 months. The baseline median knee dysfunction score was 412.5 and decreased by 108.5 over 24 months. The baseline median knee stiffness score was 54.0 and decreased by 13.5 over 24 months.

**Table 1:** Characteristics of participants

Characteristics	Values
Age^a^	63.3 ± 7.2
Female (%)^b^	46%
BMI (kg/m^2^)^a^	29.4 ± 4.9
Baseline MTF osteophytes (0–18)^c^	4.0 (2.0, 7.0)
Baseline LTF osteophytes (0–18)^c^	4.0 (2.0, 8.0)
Baseline patellar osteophytes (0–6)^c^	2.0 (2.0, 3.8)
Baseline total cartilage defects (0–24)^c^	15.0 (12.0, 18.0)
Baseline total cartilage volume (cm^3^)^c^	5.7 (4.6, 7.0)
Baseline total knee pain (0–500)^c^	115.0 (66.0, 196.8)
Change in total knee pain^c^	− 33.0 (− 95.0, 8.0)
Baseline weight-bearing pain (0–300)^c^	79.5 (42.0, 126.0)
Change in weight-bearing pain^c^	− 21.0 (− 63.0, 10.0)
Baseline non-weight-bearing pain (0–200)^c^	32.0 (73.0, 74.0)
Change in non-weight-bearing pain^c^	− 8.0 (− 44.0, 4.0)
Baseline knee dysfunction (0–1700)^c^	412.5 (218.3, 668.0)
Change in knee dysfunction^c^	− 108.5 (− 301.3, 16.3)
Baseline knee stiffness (0–200)^c^	54.0 (25.0, 92.0)
Change in knee stiffness^c^	− 13.5 (− 43.0, 8.8)

*BMI* Body mass index, *MTF* Medial tibiofemoral, *LTF* Lateral tibiofemoral

^a^Values are mean ± standard deviation

^b^Values are percentage

^c^Values are median (interquartile range)

### Mediation by osteophytes on the association between total cartilage defects and change in knee symptoms

Total cartilage defect score was significantly associated with change in WOMAC total knee pain (*β*: 3.53, 95% CI: 0.78 to 6.50), change in weight-bearing pain over 24 months (*β*: 2.05, 95% CI: 0.26 to 4.01), and change in non-weight-bearing pain over 24 months (*β*: 1.51, 95% CI: 0.21 to 2.81), after adjustment for age, sex, BMI, and vitamin D supplement. However, the total cartilage defect score had no significant association with change in stiffness and change in physical dysfunction. Causal mediation analysis showed significant mediation by osteophytes for total cartilage defect score in relation to change in total WOMAC pain and change in weight-bearing pain, after adjustment for age, sex, BMI, and vitamin D supplement. In the relation between total cartilage defect score and change in total WOMAC knee pain, 49% of the total effect was attributable to the LTF osteophytes (*P* = 0.03), and 55% of the total effect was attributable to the patellar osteophytes (*P* < 0.01). In the relation between total cartilage defect score and change in weight-bearing pain, 62% of the total effect was mediated by the LTF osteophytes (*P* = 0.02), and 61% of the total effect was mediated by the patellar osteophytes (*P* < 0.01). Forty-six percent of the total effect between the total cartilage defect score and change in non-weight-bearing pain was mediated by the patellar osteophytes (*P* < 0.01). However, the MTF osteophytes had no significant mediating effects in the relation between total cartilage defect score and changes in knee symptoms (Table 2).

**Table 2:** Mediation by osteophytes on the associations between total cartilage defects and changes in knee symptoms

Outcomes	MTF osteophyte, ***β*** (95% CI)	***P***	LTF osteophyte, ***β*** (95% CI)	***P***	Patellar osteophyte, ***β*** (95% CI)	***P***
**Change in total knee pain**						
Indirect effect	0.84 (− 0.94, 2.81)	0.36	**1.74 (0.15, 3.44)**	**0.03**	**1.95 (0.70, 3.32)**	**< 0.01**
Direct effect	2.68 (− 0.80, 6.27)	0.13	1.78 (− 1.40, 4.93)	0.28	1.58 (− 1.45, 4.76)	0.30
Total effect	**3.53 (0.53, 6.66)**	**0.02**	**3.53 (0.52, 6.34)**	**0.02**	**3.53 (0.60, 6.55)**	**0.02**
Proportion mediated %	**NA**		**49%**		**55%**	
**Change in weight-bearing pain**						
Indirect effect	0.62 (− 0.60, 1.91)	0.29	**1.26 (0.24, 2.36)**	**0.02**	**1.24 (0.45, 2.16)**	**< 0.01**
Direct effect	1.43 (− 0.72, 3.53)	0.19	0.79 (− 1.38, 2.98)	0.49	0.81 (− 1.11, 2.71)	0.41
Total effect	**2.05 (0.08, 4.06)**	**0.04**	**2.05 (0.14, 4.12)**	**0.04**	**2.05 (0.09, 3.99)**	**0.04**
Proportion mediated %	NA		**62%**		**61%**	
**Change in non-weight-bearing pain**						
Indirect effect	0.22 (− 0.56, 1.04)	0.56	0.48 (− 0.28, 1.26)	0.22	**0.70 (0.12, 1.35)**	**0.01**
Direct effect	1.29 (− 0.23, 2.84)	0.10	1.03 (− 0.50, 2.52)	0.15	0.81 (− 0.41, 2.06)	0.21
Total effect	**1.51 (0.26, 2.89)**	**0.02**	**1.51 (0.23, 2.81)**	**0.02**	**1.51 (0.36, 2.74)**	**0.01**
Proportion mediated %	NA		NA		**46%**	
**Change in stiffness**						
Indirect effect	0.35 (− 0.40, 1.11)	0.36	**1.17 (0.35, 2.09)**	**< 0.01**	**0.81 (0.26, 1.51)**	**< 0.01**
Direct effect	0.17 (− 1.35, 1.75)	0.82	− 0.65 (− 2.25, 0.80)	0.39	− 0.30 (− 1.80, 1.11)	0.67
Total effect	0.52 (− 0.86, 1.93)	0.45	0.52 (− 0.80, 1.88)	0.46	0.52 (− 0.90, 1.91)	0.50
Proportion mediated %	NA		NA		NA	
**Change in dysfunction**						
Indirect effect	4.22 (− 0.90, 9.55)	0.10	**6.63 (1.17, 11.90)**	**0.02**	**5.19 (1.46, 9.91)**	**< 0.01**
Direct effect	2.74 (− 6.08, 11.64)	0.56	0.33 (− 8.07, 9.99)	0.92	1.77 (− 6.74, 10.03)	0.69
Total effect	6.96 (− 1.78, 15.72)	0.11	6.96 (− 0.72, 16.12)	0.09	6.96 (− 1.22, 15.71)	0.10
Proportion mediated %	NA		NA		NA	

Adjusted for age, sex, BMI, and vitamin D supplement

Statistically significant associations are shown in bold

*MTF* Medial tibiofemoral, *LTF* Lateral tibiofemoral

### Mediation by osteophytes on the association between total cartilage volume and change in knee symptoms

Total cartilage volume was significantly associated with change in weight-bearing pain over 24 months (*β*: − 6.49, 95% CI: − 12.71 to − 0.27) and change in physical dysfunction over 24 months (*β*: − 32.29, 95% CI: − 59.74 to − 4.84), after adjustment for age, sex, BMI, and vitamin D supplement. However, total cartilage volume had no significant association with change in total WOMAC knee pain, change in non-weight-bearing pain, or change in stiffness. Causal mediation analysis showed significant mediation by osteophytes for total cartilage volume in relation to change in weight-bearing pain and change in physical dysfunction after adjustment for age, sex, BMI, and vitamin D supplement (Table 3). In the relation between total cartilage volume and change in weight-bearing pain, 27% of the total effect was attributable to the LTF osteophytes (*P* < 0.01), and 30% of the total effect was attributable to the patellar osteophytes (*P* < 0.01). In the relation between total cartilage volume and change in physical dysfunction, 25% of the total effect was attributable to the LTF osteophytes (*P* < 0.01), and 24% of the total effect was attributable to the patellar osteophytes (*P* < 0.01). However, the MTF osteophytes had no significant mediating effect on the relation between total cartilage volume and change in knee symptoms.

**Table 3:** Mediation by osteophytes on the associations between total cartilage volume and changes in knee symptoms

Outcomes	MTF osteophyte, ***β*** (95% CI)	***P***	LTF osteophyte, ***β*** (95% CI)	***P***	Patellar osteophyte, ***β*** (95% CI)	***P***
**Change in total knee pain**						
Indirect effect	− 1.64 (− 4.17, 0.25)	0.10	**− 2.52 (− 5.24, − 0.69)**	**< 0.01**	**− 3.16 (− 6.29, − 0.97)**	**< 0.01**
Direct effect	− 4.66 (− 14.13, 4.17)	0.29	− 3.79 (− 13.66, 5.77)	0.44	− 3.14 (− 12.55, 6.18)	0.49
Total effect	− 6.30 (− 15.81, 2.56)	0.16	− 6.30 (− 16.31, 3.29)	0.19	− 6.30 (− 15.86, 2.99)	0.17
Proportion mediated %	NA		NA		NA	
**Change in weight-bearing pain**						
Indirect effect	− 1.04 (− 2.71, 0.13)	0.09	**− 1.74 (− 3.49, − 0.48)**	**< 0.01**	**− 1.93 (− 4.04, − 0.48)**	**< 0.01**
Direct effect	− 5.45 (− 12.06, 1.04)	0.10	− 4.75 (− 11.34, 1.31)	0.13	− 4.56 (− 11.03, 1.60)	0.15
Total effect	**− 6.49 (− 13.45, − 0.11)**	**0.04**	**− 6.49 (− 13.54, − 0.33)**	**0.04**	**− 6.49 (− 13.18, − 0.31)**	**0.04**
Proportion mediated %	NA		**27%**		**30%**	
**Change in non-weight-bearing pain**						
Indirect effect	− 0.67 (− 1.72, 0.08)	0.09	**− 0.96 (− 2.09, − 0.17)**	**0.02**	**− 1.26 (− 2.61, − 0.27)**	**< 0.01**
Direct effect	− 0.56 (− 4.73, 3.85)	0.81	− 0.28 (− 4.54, 3.92)	0.88	0.02 (− 4.26, 4.10)	0.99
Total effect	− 1.24 (− 5.40, 2.97)	0.59	− 1.24 (− 5.64, 3.00)	0.53	− 1.24 (− 5.42, 2.85)	0.54
Proportion mediated %	NA		NA		NA	
**Change in stiffness**						
Indirect effect	− 0.38 (− 1.32, 0.39)	0.33	**− 1.23 (− 2.48, − 0.28)**	**< 0.01**	**− 1.16 (− 2.57, − 0.23)**	**< 0.01**
Direct effect	− 2.56 (− 6.91, 1.97)	0.25	− 1.71 (− 6.07, 2.47)	0.43	− 1.78 (− 6.07, 2.65)	0.45
Total effect	− 2.94 (− 7.28, 1.54)	0.19	− 2.94 (− 7.56, 1.45)	0.19	− 2.94 (− 7.20, 1.40)	0.19
Proportion mediated %	NA		NA		NA	
**Change in dysfunction**						
Indirect effect	− 4.85 (− 12.25, 0.57)	0.08	**− 8.22 (− 17.18, − 2.17)**	**< 0.01**	**− 7.87 (− 17.80, − 1.39)**	**< 0.01**
Direct effect	**− 27.44 (− 54.13, − 1.47)**	**0.04**	− 24.08 (− 49.24, 3.49)	0.08	− 24.42 (− 49.60, 1.95)	0.07
Total effect	**− 32.29 (− 59.92, − 6.22)**	**0.02**	**− 32.29 (− 58.16, − 5.31)**	**0.02**	**− 32.29 (− 58.03, − 5.63)**	**0.01**
Proportion mediated %	NA		**25%**		**24%**	

Adjusted for age, sex, BMI, and vitamin D supplement

Statistically significant associations are shown in bold

*MTF* Medial tibiofemoral, *LTF* Lateral tibiofemoral

Follow-up patellar osteophytes significantly mediated the associations between baseline total cartilage defects and change in knee pain (28–31%). However, follow-up LTF osteophytes only had borderline mediating effects on the association between baseline total cartilage defects and change in knee symptoms. In addition, LTF osteophytes and patellar osteophytes significantly mediated the associations between baseline total cartilage volumes and change in weight-bearing pain/physical dysfunction (17–25%) (Additional file 1: Tables S10-S11).

## Discussion

In participants with symptomatic knee OA, we found that the significant associations between cartilage morphology and change in knee symptoms were indirect. The associations between total cartilage defect score and change in total WOMAC knee pain and change in weight-bearing pain were partly mediated by osteophytes in the LTF and patellar compartments. The associations between total cartilage volume and change in weight-bearing pain and change in physical dysfunction were partly mediated by osteophytes in the LTF and patellar compartments. These findings indicated that cartilage may not be the direct cause of osteoarthritic symptoms, and osteophyte formation was an important mediator of this process.

To our knowledge, this is the first study to reveal that the associations between cartilage morphology and change in knee symptoms are partly mediated via osteophytes. For a long time, studies on the structural causes of osteoarthritic knee symptoms have been focused on articular cartilage [31]. Hunter et al. reported that patellar cartilage volume had a strongly inverse relationship with WOMAC pain and WOMAC dysfunction [32]. A brief report from FNIH OA Biomarkers Consortium found that cartilage thickness loss was associated with pain progression [5]. In a study with 500 participants, the prevalence and severity of knee pain were significantly associated with medial tibial cartilage defects [33]. In addition, there was a dose-response relationship between knee pain and number of sites having grade 3 or 4 cartilage defects, with all participants having knee pain if all compartments of the knee had these defects [33]. A population-based study with 2733 participants reported that joint space narrowing (a proxy for cartilage loss) had a significant association with knee pain [34]. These previous studies found the association between cartilage morphology and knee symptoms, but there were few studies to explore how they were related. Our findings indicated that cartilage was not the direct source of knee pain and knee dysfunction but indirectly through osteophytes. Weight-bearing pain is our primary measurement because osteoarthritic knee pain is typically intermittent and mainly weight-bearing (mechanical) pain [2]. A study from OAI reported that osteophytes were associated with weight-bearing pain but not non-weight-bearing pain after adjustment [35]. Our findings supported these results and revealed that osteophytes mediated the associations between cartilage morphology and change in weight-bearing pain, but not the association between cartilage morphology and change in non-weight-bearing pain, indicating the pain associated with osteophytes is mainly mechanical. Pain is a major driver of clinical decision-making, and our findings have some implications in clinical practice. Foremost, the indirect associations and the large proportion of mediating effects between cartilage morphology and knee symptoms could help clinicians to choose the appropriate treatment target abnormalities according to MRI images. In addition, our findings could help physicians to better predict patients’ pain progression from MRI images because doctors could combine various lesions to make a comprehensive judgment.

Many trials have used cartilage morphology as the main outcome of treatment and hope to delay the progression of symptoms by reducing cartilage loss. In two recent clinical trials, both sprifermin (fibroblast growth factor 18 agonist) and MIV-711 (selective cathepsin K inhibitor) can significantly improve cartilage thickness, but they had no effect on knee symptoms [36, 37]. The indirect association between cartilage morphology and knee symptoms identified in our study may explain why treatments targeting cartilage have had no significant effect on knee pain. Both clinical trials included participants with established OA (Kellgren-Lawrence grade 2 or 3), which means severe osteophytes were already present. The indirect effect of chondroprotective treatment on pain may be limited due to the population being assessed having established osteoarthritis and severe osteophytes. A recent study also reported that the effect of cartilage loss on knee symptom progression was not direct but mediated by synovitis. However, the reported mediating effect of synovitis was only 14.11% over 2 years [9]. We also analyzed the mediating effects of effusion-synovitis and BMLs on the associations between cartilage morphology and knee symptoms, which showed no significant mediating effects (data not shown). These results are not consistent with a previous study using the OAI database [9]. The inconsistency may own to a smaller sample size and different MRI scoring systems in the current VIDEO database.

Our study found a more important mediator between cartilage and knee pain, and the mediating effects of osteophytes were 24–62%. Interestingly, we found that only LTF osteophytes and patellar osteophytes had mediating effects on the relationship between cartilage morphology and change in knee symptoms. Gaine et al. reported that lateral osteophyte impingement of the popliteus tendon could be a direct cause of lateral knee joint pain and dysfunction [38]. We assume that the association between LTF osteophytes and weight-bearing pain and dysfunction may be caused by the popliteus tendon impinged during flexion. In addition, cartilage morphology may not be the major cause of MTF osteophytes. Other structural abnormalities such as medial meniscal extrusion have been reported to be the most closely associated structural abnormality with osteophyte formation [39]. However, the mechanism of osteophytes on pain still warrants further investigations. According to the existing literature, patellofemoral OA is the most common cause of anterior knee pain [40]. The WOMAC is an important tool for assessing anterior knee pain [41, 42], which may explain why patellar osteophytes mediated the association between cartilage morphology and change in WOMAC pain. In the subgroup analysis stratified by vitamin D intervention or not, there were no significant associations between cartilage morphology and change in knee symptoms in the vitamin D group. Moreover, the significant mediating effects became borderline significant in the placebo group. This may be due to the reduction of sample size and reduced statistical power (Additional file 1: Tables S1-S4). In the subgroup analysis stratified by gender, there were no significant associations between cartilage morphology and change in knee symptoms in females, which may be due to the reduction in the sample size and lower statistical power. However, in males, osteophytes mediated the associations of cartilage volumes with a change in weight-bearing pain (50–78%) (Additional file 1: Table S6). This gender disparity suggests that there is a higher proportion of mediating effects of osteophytes in males than in females.

One possible mechanism for the mediating effect of osteophytes on the relationship between cartilage morphology and change in knee symptoms is that cartilage lesions promote the formation of osteophytes and osteophytes can induce knee symptoms. The development of osteophytes is considered an adaptive response of the damaged knee in an attempt to maintain joint balance [43]. The formation of osteophytes could be activated by damaged cartilage to maintain joint stability, but severe osteophytes could be related to knee pain and limit physical function. The TGF-β signal pathway has been related to the formation of osteophytes [44] via bone morphogenetic protein (BMP). BMP-2 is barely present in normal adult articular cartilage but is present in moderately or severely damaged OA cartilage [45]. In transgenic mice, elevated BMPs specifically in chondrocytes exacerbated the formation of osteophytes [46], which indicated that as a secretory protein, the increase of BMP-2 in damaged cartilage may induce osteophyte formation. Osteophytes are rich in nerve fibers, making them an important source of knee pain [10]. Our results suggested that osteophytes may be responsible for nociceptive stimuli in OA but not cartilage itself, since it does not contain nerve fibers and therefore cannot directly generate algesia.

The strength of our study was using MRI to detect cartilage volume, cartilage defects, and osteophytes. Some limitations of our study should be considered. First, the original study only included participants with symptomatic knee OA, so these findings may not be generalized to early-stage disease. Second, our results may be affected by the interventions of the original clinical trial, but we have adjusted for interventions in multi-variable analyses, indicating the impact of the intervention is minimal. Last, we did not perform adjustments for potential multiplicity. This is because exploratory studies usually do not include typically data-generated hypotheses, which is thought to be unnecessary for multiplicity corrections. However, as an exploratory study, our results need to be interpreted with caution. Further experimental studies are needed to confirm our findings.

## Conclusions

The significant associations between cartilage morphology and changes in knee symptoms were indirect, and the associations were partly mediated by osteophytes.

## Supplementary Information

**Additional file 1: Table S1.** Mediation by osteophytes on the associations between total cartilage defects and changes in knee symptoms in vitamin D supplement group. **Table S2.** Mediation by osteophytes on the associations between total cartilage defects and changes in knee symptoms in placebo group. **Table S3.** Mediation by osteophytes on the associations between total cartilage volumes and changes in knee symptoms in vitamin D supplement group. **Table S4.** Mediation by osteophytes on the associations between total cartilage volumes and changes in knee symptoms in placebo group. **Table S5.** Baseline characteristics of participants who completed the study vs loss to follow-up. **Table S6.** Mediation by osteophytes on the associations between total cartilage defects and changes in the knee symptoms in males. **Table S7.** Mediation by osteophytes on the associations between total cartilage defects and changes in knee symptoms in females. **Table S8.** Mediation by osteophytes on the associations between total cartilage volumes and changes in knee symptoms in males. **Table S9.** Mediation by osteophytes on the associations between total cartilage volumes and changes in knee symptoms in females. **Table S10.** Mediation by follow-up osteophytes on the associations between baseline total cartilage defects and changes in knee symptoms. **Table S11.** Mediation by follow-up osteophytes on the associations between baseline total cartilage volume and changes in knee symptoms.

## Abbreviations

BMI: Body mass index
BMLs: Bone marrow lesions
ICCs: Intra-class correlation coefficients
KOSS: Knee Osteoarthritis Scoring System
LTF: Lateral tibiofemoral
MTF: Medial tibiofemoral
MRI: Magnetic resonance imaging
OA: Osteoarthritis
VIDEO: Vitamin D Effect on Osteoarthritis
WOMAC: Western Ontario and McMaster Universities Osteoarthritis Index
WORMS: Whole-Organ Magnetic Resonance Imaging Score
