# Supplementary material for: Osteophytes mediate the associations between cartilage morphology and changes in knee symptoms in patients with knee osteoarthritis
Source: Arthritis Res Ther. 2022 Sep 8;24:217. doi: 10.1186/s13075-022-02905-8 (PMC9454107; doi:10.1186/s13075-022-02905-8)
Supplement: Supplementary file 1 — Additional file 1: Table S1. Mediation by osteophytes on the associations between total cartilage defects and changes in knee symptoms in vitamin D supplement group. Table S2. Mediation by osteophytes on the associations between total cartilage defects and changes in knee symptoms in placebo group. Table S3. Mediation by osteophytes on the associations between total cartilage volumes and changes in knee symptoms in vitamin D supplement group. Table S4. Mediation by osteophytes on the associations between total cartilage volumes and changes in knee symptoms in placebo group. Table S5. Baseline characteristics of participants who completed the study vs loss to follow-up. Table S6. Mediation by osteophytes on the associations between total cartilage defects and changes in the knee symptoms in males. Table S7. Mediation by osteophytes on the associations between total cartilage defects and changes in knee symptoms in females. Table S8. Mediation by osteophytes on the associations between total cartilage volumes and changes in knee symptoms in males. Table S9. Mediation by osteophytes on the associations between total cartilage volumes and changes in knee symptoms in females. Table S10. Mediation by follow-up osteophytes on the associations between baseline total cartilage defects and changes in knee symptoms. Table S11. Mediation by follow-up osteophytes on the associations between baseline total cartilage volume and changes in knee symptoms. [file 13075_2022_2905_MOESM1_ESM.docx]

eTable 1. Mediation by osteophytes on the associations between total cartilage defects and changes in knee symptoms in vitamin D supplement group

| **Outcomes** | **MTF osteophyte**  **β (95% CI)** | ***P*** | **LTF osteophyte**  **β (95% CI)** | ***P*** | **Patellar osteophyte**  **β (95% CI)** | ***P*** |
| --- | --- | --- | --- | --- | --- | --- |
| **Change in total knee pain** |  |  |  |  |  |  |
| Indirect effect | 2.25 (-0.24, 5.10) | 0.09 | **2.18 (0.03, 4.57)** | **<0.05** | 1.57 (-0.42, 3.64) | 0.11 |
| Direct effect | -0.35 (-5.24, 4.73) | 0.93 | -0.28 (-4.80, 4.28) | 0.902 | 0.33 (-3.97, 4.78) | 0.86 |
| Total effect | 1.90 (-1.89, 6.09) | 0.31 | 1.90 (-2.13, 5.98) | 0.344 | 1.90 (-1.96, 6.13) | 0.35 |
| Proportion mediated% | NA |  | NA |  | NA |  |
| **Change in weight-bearing pain** |  |  |  |  |  |  |
| Indirect effect | 1.43 (-0.01, 3.18) | 0.05 | 1.27 (-0.13, 2.82) | 0.07 | 0.64 (-0.60, 2.07) | 0.33 |
| Direct effect | -0.56 (-3.35, 2.25) | 0.68 | -0.40 (-3.28, 2.46) | 0.80 | 0.23 (-2.33, 2.89) | 0.89 |
| Total effect | 0.87 (-1.50, 3.34) | 0.48 | 0.87 (-1.56, 3.41) | 0.50 | 0.87 (-1.60, 3.44) | 0.53 |
| Proportion mediated% | NA |  | NA |  | NA |  |
| **Change in non-weight-bearing pain** |  |  |  |  |  |  |
| Indirect effect | 0.82 (-0.43, 2.16) | 0.21 | 0.91 (-0.34, 2.10) | 0.13 | **0.93 (0.01, 2.02)** | **< 0.05** |
| Direct effect | 0.21 (-2.19, 2.67) | 0.84 | 0.12 (-2.03, 2.37) | 0.91 | 0.10 (-1.88, 2.11) | 0.91 |
| Total effect | 1.03 (-0.79, 3.11) | 0.28 | 1.03 (-0.86, 2.91) | 0.27 | 1.03 (-0.71, 3.00) | 0.25 |
| Proportion mediated% | NA |  | NA |  | NA |  |
| **Change in stiffness** |  |  |  |  |  |  |
| Indirect effect | 0.29 (-0.88, 1.52) | 0.61 | **1.36 (0.06, 2.73)** | **0.04** | 0.87 (-0.16, 2.07) | 0.08 |
| Direct effect | 0.19 (-2.07, 2.46) | 0.86 | -0.88 (-3.16, 1.54) | 0.50 | -0.38 (-2.54, 1.67) | 0.75 |
| Total effect | 0.49 (-1.45, 2.49) | 0.62 | 0.49 (-1.35, 2.44) | 0.63 | 0.49 (-1.37, 2.38) | 0.59 |
| Proportion mediated% | NA |  | NA |  | NA |  |
| **Change in dysfunction** |  |  |  |  |  |  |
| Indirect effect | **6.80 (0.01, 15.14)** | **<0.05** | **9.94 (1.55, 19.55)** | **0.02** | 4.73 (-1.51, 12.0) | 0.15 |
| Direct effect | -4.51 (-18.48, 8.41) | 0.50 | -7.65 (-21.20, 5.54) | 0.24 | -2.44 (-15.61, 12.0) | 0.68 |
| Total effect | 2.29 (-9.65, 14.01) | 0.69 | 2.29 (-9.25, 14.03) | 0.73 | 2.29 (-9.58, 14.2) | 0.70 |
| Proportion mediated% | NA |  | NA |  | NA |  |

Adjusted for age, sex and body mass index.

Statistically significant associations are shown in bold.

MTF, medial tibiofemoral; LTF, lateral tibiofemoral.

eTable 2. Mediation by osteophytes on the associations between total cartilage defects and changes in knee symptoms in placebo group

| **Outcomes** | **MTF osteophyte**  **β (95% CI)** | ***P*** | **LTF osteophyte**  **β (95% CI)** | ***P*** | **Patellar osteophyte**  **β (95% CI)** | ***P*** |
| --- | --- | --- | --- | --- | --- | --- |
| **Change in total knee pain** |  |  |  |  |  |  |
| Indirect effect | -0.40 (-3.23, 2.48) | 0.81 | 1.44 (-0.75, 4.05) | 0.20 | **2.17 (0.60, 4.41)** | **<0.01** |
| Direct effect | **5.73 (0.54, 11.32)** | **0.02** | 3.89 (-1.27, 8.55) | 0.13 | 3.15 (-0.91, 7.41) | 0.13 |
| Total effect | **5.33 (0.97, 10.08)** | **0.02** | **5.33 (0.55, 9.79)** | **0.03** | **5.33 (0.87, 10.18)** | **0.02** |
| Proportion mediated% | NA |  | NA |  | **41%** |  |
| **Change in weight-bearing pain** |  |  |  |  |  |  |
| Indirect effect | -0.10 (-2.04, 1.94) | 0.90 | 1.37 (-0.29, 3.05) | 0.11 | **1.63 (0.58, 3.06)** | **<0.01** |
| Direct effect | **3.51 (0.03, 6.92)** | **<0.05** | 2.04 (-1.34, 5.42) | 0.23 | 1.78 (-1.07, 4.69) | 0.23 |
| Total effect | **3.41 (0.45, 6.54)** | **0.02** | **3.41 (0.40, 6.70)** | **0.03** | **3.41 (0.33, 6.54)** | **0.03** |
| Proportion mediated% | NA |  | NA |  | **48%** |  |
| **Change in non-weight-bearing pain** |  |  |  |  |  |  |
| Indirect effect | -0.30 (-1.45, 0.74) | 0.60 | 0.07 (-0.93, 1.16) | 0.94 | 0.54 (-0.13, 1.39) | 0.10 |
| Direct effect | **2.22 (0.29, 4.20)** | **0.02** | 1.85 (-0.04, 3.78) | 0.06 | 1.37 (-0.31, 3.14) | 0.11 |
| Total effect | **1.91 (0.22, 3.69)** | **0.03** | **1.91 (0.15, 3.79)** | **0.03** | **1.91 (0.24, 3.73)** | **0.03** |
| Proportion mediated% | NA |  | NA |  | NA |  |
| **Change in stiffness** |  |  |  |  |  |  |
| Indirect effect | 0.45 (-0.55, 1.58) | 0.38 | **1.21 (0.12, 2.41)** | **0.03** | **0.76 (0.16, 1.54)** | **0.01** |
| Direct effect | 0.17 (-2.12, 2.36) | 0.93 | -0.60 (-2.63, 1.55) | 0.57 | -0.14 (-2.04, 1.82) | 0.89 |
| Total effect | 0.62 (-1.32, 2.56) | 0.57 | 0.62 (-1.23, 2.67) | 0.50 | 0.62 (-1.34, 2.59) | 0.52 |
| Proportion mediated% | NA |  | NA |  | NA |  |
| **Change in dysfunction** |  |  |  |  |  |  |
| Indirect effect | 1.97 (-5.13, 9.87) | 0.55 | 4.63 (-2.52, 12.64) | 0.21 | **5.50 (1.07, 11.85)** | **0.01** |
| Direct effect | 9.43 (-3.33, 21.76) | 0.14 | 6.77 (-5.88, 19.18) | 0.28 | 5.90 (-4.92, 17.03) | 0.29 |
| Total effect | 11.40 (-0.23, 23.68) | 0.06 | 11.40 (-0.59, 24.36) | 0.07 | 11.40 (-0.25, 23.90) | 0.06 |
| Proportion mediated% | NA |  | NA |  | NA |  |

Adjusted for age, sex, and body mass index.

Statistically significant associations are shown in bold.

MTF, medial tibiofemoral; LTF, lateral tibiofemoral.

eTable 3. Mediation by osteophytes on the associations between total cartilage volumes and changes in knee symptoms in vitamin D supplement group

| **Outcomes** | **MTF osteophyte**  **β (95% CI)** | ***P*** | **LTF osteophyte**  **β (95% CI)** | ***P*** | **Patellar osteophyte**  **β (95% CI)** | ***P*** |
| --- | --- | --- | --- | --- | --- | --- |
| **Change in total knee pain** |  |  |  |  |  |  |
| Indirect effect | -2.12 (-5.90, 0.17) | 0.08 | -1.13 (-4.07, 0.99) | 0.29 | -2.00 (-5.90, 0.16) | 0.09 |
| Direct effect | 3.47 (-8.14, 16.23) | 0.60 | 2.48 (-10.66, 15.64) | 0.72 | 3.35 (-8.95, 15.95) | 0.60 |
| Total effect | 1.35 (-10.18, 13.72) | 0.87 | 1.35 (-11.45, 13.85) | 0.85 | 1.35 (-10.77, 13.75) | 0.82 |
| Proportion mediated% | NA |  | NA |  | NA |  |
| **Change in weight-bearing pain** |  |  |  |  |  |  |
| Indirect effect | -1.20 (-3.42, 0.22) | 0.12 | -0.60 (-2.29, 0.59) | 0.31 | -0.77 (-3.13, 0.62) | 0.27 |
| Direct effect | -0.50 (-9.37, 7.79) | 0.86 | -1.09 (-9.35, 6.57) | 0.75 | -0.92 (-9.77, 7.33) | 0.79 |
| Total effect | -1.70 (-10.31, 6.47) | 0.66 | -1.70 (-9.90, 5.71) | 0.63 | -1.70 (-10.37, 6.10) | 0.65 |
| Proportion mediated% | NA |  | NA |  | NA |  |
| **Change in non-weight-bearing pain** |  |  |  |  |  |  |
| Indirect effect | -0.92 (-2.54, 0.12) | 0.10 | -0.53 (-1.83, 0.45) | 0.29 | **-1.23 (-3.09, 0.01)** | **0.05** |
| Direct effect | 3.96 (-2.04, 10.33) | 0.19 | 3.57 (-2.38, 9.30) | 0.22 | 4.27 (-1.81, 10.11) | 0.17 |
| Total effect | 3.05 (-2.83, 9.16) | 0.29 | 3.05 (-2.82, 8.79) | 0.30 | 3.05 (-2.85, 8.78) | 0.34 |
| Proportion mediated% | NA |  | NA |  | NA |  |
| **Change in stiffness** |  |  |  |  |  |  |
| Indirect effect | -0.31 (-1.56, 0.93) | 0.56 | -0.59 (-2.07, 0.49) | 0.31 | -0.92 (-3.09, 0.11) | 0.10 |
| Direct effect | -0.68 (-6.85, 5.60) | 0.79 | -0.41 (-6.59, 5.62) | 0.90 | -0.07 (-6.26, 6.60) | 0.97 |
| Total effect | -0.99 (-7.13, 5.37) | 0.73 | -0.99 (-7.22, 5.19) | 0.76 | -0.99 (-6.87, 5.19) | 0.78 |
| Proportion mediated% | NA |  | NA |  | NA |  |
| **Change in dysfunction** |  |  |  |  |  |  |
| Indirect effect | -5.21 (-14.81, 1.35) | 0.15 | -4.07 (-14.57, 3.35) | 0.30 | -4.82 (-16.36, 1.81) | 0.18 |
| Direct effect | -4.83 (-43.23, 35.29) | 0.80 | -5.96 (-42.62, 33.32) | 0.75 | -5.22 (-43.22, 33.81) | 0.78 |
| Total effect | -10.04 (-47.75, 27.71) | 0.60 | -10.04 (-48.32, 29.09) | 0.59 | -10.04 (-47.73, 27.01) | 0.56 |
| Proportion mediated% | NA |  | NA |  | NA |  |

Adjusted for age, sex and body mass index.

Statistically significant associations are shown in bold.

MTF, medial tibiofemoral; LTF, lateral tibiofemoral.

eTable 4. Mediation by osteophytes on the associations between total cartilage volumes and changes in knee symptoms in placebo group

| **Outcomes** | **MTF osteophyte**  **β (95% CI)** | ***P*** | **LTF osteophyte**  **β (95% CI)** | ***P*** | **Patellar osteophyte**  **β (95% CI)** | ***P*** |
| --- | --- | --- | --- | --- | --- | --- |
| **Change in total knee pain** |  |  |  |  |  |  |
| Indirect effect | -0.81 (-5.06, 2.76) | 0.59 | -3.72 (-8.93, 0.76) | 0.10 | **-4.37 (-9.92, -0.40)** | **0.02** |
| Direct effect | **-14.32 (-28.89, -0.56)** | **0.04** | -11.41 (-25.80, 2.62) | 0.10 | -10.76 (-23.73, 2.68) | 0.11 |
| Total effect | **-15.13 (-30.30, -1.72)** | **0.03** | **-15.13 (-29.43, -1.17)** | **0.04** | **-15.13 (-29.44, -1.01)** | **0.04** |
| Proportion mediated% | NA |  | NA |  | **29%** |  |
| **Change in weight-bearing pain** |  |  |  |  |  |  |
| Indirect effect | -0.64 (-3.55, 1.51) | 0.55 | **-2.97 (-6.47, -0.20)** | **0.04** | **-3.19 (-7.22, -0.43)** | **0.02** |
| Direct effect | -9.99 (-20.12, 0.05) | 0.05 | -7.66 (-17.61, 1.83) | 0.11 | -7.44 (-16.72, 1.67) | 0.10 |
| Total effect | **-10.63 (-20.42, -0.27)** | **0.04** | **-10.63 (-20.96, -1.04)** | **0.04** | -10.63 (-21.25, -0.79) | **0.03** |
| Proportion mediated% | NA |  | **28%** |  | **30%** |  |
| **Change in non-weight-bearing pain** |  |  |  |  |  |  |
| Indirect effect | -0.17 (-1.59, 1.17) | 0.76 | -0.75 (-2.90, 1.06) | 0.44 | -1.18 (-3.40, 0.06) | 0.08 |
| Direct effect | -4.33 (-10.47, 1.61) | 0.14 | -3.75 (-9.71, 1.47) | 0.17 | -3.32 (-8.75, 1.98) | 0.24 |
| Total effect | -4.50 (-10.48, 1.02) | 0.11 | -4.50 (-10.20, 0.56) | 0.08 | -4.50 (-10.09, 0.84) | 0.10 |
| Proportion mediated% | NA |  | NA |  | NA |  |
| **Change in stiffness** |  |  |  |  |  |  |
| Indirect effect | -0.54 (-2.07, 0.59) | 0.40 | -1.86 (-4.23, 0.05) | 0.06 | **-1.35 (-3.28, -0.07)** | **0.03** |
| Direct effect | -2.68 (-9.42, 3.65) | 0.36 | -1.36 (-7.43, 4.34) | 0.66 | -1.87 (-7.38, 3.91) | 0.53 |
| Total effect | -3.22 (-9.91, 3.10) | 0.28 | -3.22 (-9.67, 2.95) | 0.30 | -3.22 (-9.37, 3.02) | 0.32 |
| Proportion mediated% | NA |  | NA |  | NA |  |
| **Change in dysfunction** |  |  |  |  |  |  |
| Indirect effect | -4.16 (-16.70, 3.89) | 0.35 | -9.20 (-25.69, 3.85) | 0.17 | **-10.50 (-26.90, -0.55)** | **0.03** |
| Direct effect | **-39.10 (-78.56, -2.01)** | **0.04** | -34.06 (-75.51, 3.43) | 0.07 | -32.80 (-66.80, 3.47) | 0.09 |
| Total effect | **-43.26 (-82.59, -7.13)** | **0.01** | **-43 (-84.97, -6.73)** | **0.02** | **-43.30 (-81.50, -6.07)** | **0.02** |
| Proportion mediated% | NA |  | NA |  | **24%** |  |

Adjusted for age, sex and body mass index.

Statistically significant associations are shown in bold.

MTF, medial tibiofemoral; LTF, lateral tibiofemoral.

eTable 5. Baseline characteristics of participants who completed the study vs loss to follow-up

|  | Completed | Loss to follow-up |  |
| --- | --- | --- | --- |
|  | 334 | 73 | P-value |
| Age^a^ | 63.3 ± 7.2 | 63.1 ± 7.0 | 0.89 |
| Female (%)^b^ | 46% | 68% | **<0.01** |
| BMI (kg/m²)^a^ | 29.4 ± 4.9 | 30.0 ± 5.7 | 0.41 |
| Baseline MTF osteophytes (0-18)^c^ | 4.0 (2.0, 7.0) | 4.0 (2.0, 7.3) | 0.80 |
| Baseline LTF osteophytes (0-18)^c^ | 4.0 (2.0, 8.0) | 4.0 (2.0, 8.0) | 0.55 |
| Baseline patellar osteophytes (0-6)^c^ | 2.0 (2.0, 3.8) | 2.0 (2.0, 4.0) | 0.93 |
| Baseline total cartilage defects (0-24)^c^ | 15.0 (12.0, 18.0) | 13.0 (11.0, 17.0) | 0.14 |
| Baseline total cartilage volume (cm³)^c^ | 5.7 (4.6, 7.0) | 4.72 (4.10, 5.90) | **<0.01** |
| Baseline total knee pain (0-500) ^c^ | 115.0 (66.0, 196.8) | 145.5 (88.8, 191.0) | 0.12 |
| Baseline weight-bearing pain (0-300) ^c^ | 79.5 (42.0, 126.0) | 98.5 (54.0, 142.8) | 0.09 |
| Baseline non-weight-bearing pain (0-200)^c^ | 32.0 (73.0, 74.0) | 37.5 (14.8, 74.3) | 0.22 |
| Baseline knee dysfunction (0-1700)^c^ | 412.5 (218.3, 668.0) | 502.8 (265.4, 702.8) | 0.15 |
| Baseline knee stiffness (0-200)^c^ | 54.0 (25.0, 92.0) | 56.0 (25.0, 92.5) | 0.91 |

^a^ Values are mean ± standard deviation.

^b^ Values are percentage.

^c^ Values are median (interquartile range).

Student t-test, Chi^2^ or nonparametric test was used for the comparison

BMI, body mass index; LTF, MTF, medial tibiofemoral; lateral tibiofemoral.

eTable 6. Mediation by osteophytes on the associations between total cartilage defects and changes in the knee symptoms in males

| **Outcomes** | **MTF osteophyte**  **β (95% CI)** | ***P*** | **LTF osteophyte**  **β (95% CI)** | ***P*** | **Patellar osteophyte**  **β (95% CI)** | ***P*** |
| --- | --- | --- | --- | --- | --- | --- |
| **Change in total knee pain** |  |  |  |  |  |  |
| Indirect effect | **3.01 (0.87, 5.78)** | **<0.01** | **2.84 (0.97, 5.06)** | **<0.01** | **1.97 (0.68, 3.76)** | **<0.01** |
| Direct effect | 0.26 (-4.21, 4.97) | 0.92 | 0.42 (-3.54, 4.67) | 0.84 | 1.29 (-2.37, 5.11) | 0.50 |
| Total effect | 3.26 (-0.42, 7.50) | 0.09 | 3.26 (-0.42, 7.37) | 0.08 | 3.26 (-0.50, 7.30) | 0.09 |
| Proportion mediated% | NA |  | NA |  | NA |  |
| **Change in weight-bearing pain** |  |  |  |  |  |  |
| Indirect effect | **1.97 (0.38, 3.88)** | **0.02** | **1.88 (0.63, 3.36)** | **<0.01** | **1.26 (0.37, 2.46)** | **<0.01** |
| Direct effect | 0.55 (-2.38, 3.44) | 0.66 | 0.65 (-2.13, 3.47) | 0.65 | 1.26 (-1.03, 3.85) | 0.32 |
| Total effect | **2.53 (0.12, 5.18)** | **0.04** | **2.53 (0.04, 5.39)** | **<0.05** | **2.53 (0.04, 5.34)** | **<0.05** |
| Proportion mediated% | **78%** |  | **74%** |  | **50%** |  |
| **Change in non-weight-bearing pain** |  |  |  |  |  |  |
| Indirect effect | **1.03 (0.23, 2.02)** | **0.01** | **0.96 (0.13, 1.98)** | **0.02** | **0.71 (0.18, 1.40)** | **<0.01** |
| Direct effect | -0.30 (-2.22, 1.62) | 0.79 | -0.23 (-2.12, 1.62) | 0.81 | 0.02 (-0.60, 1.67) | 0.99 |
| Total effect | 0.74 (-0.79, 2.35) | 0.33 | 0.74 (-0.77, 2.28) | 0.35 | 0.74 (-0.74, 2.35) | 0.35 |
| Proportion mediated% | NA |  | NA |  | NA |  |
| **Change in stiffness** |  |  |  |  |  |  |
| Indirect effect | 0.79 (-0.03, 1.82) | 0.06 | **1.48 (0.58, 2.51)** | **<0.01** | **0.64 (0.06, 1.41)** | **0.04** |
| Direct effect | -0.13 (-2.21, 2.11) | 0.93 | -0.81 (-2.80, 1.42) | 0.48 | 0.02 (-1.91, 2.08) | 0.95 |
| Total effect | 0.66 (-1.25, 2.84) | 0.47 | 0.66 (-1.20, 2.81) | 0.46 | 0.66 (-1.18, 2.63) | 0.47 |
| Proportion mediated% | NA |  | NA |  | NA |  |
| **Change in dysfunction** |  |  |  |  |  |  |
| Indirect effect | **7.11 (1.35, 13.85)** | **0.02** | **9.22 (3.59, 15.49)** | **<0.01** | **4.04 (0.34, 9.89)** | **0.03** |
| Direct effect | -0.68 (-12.15, 11.17) | 0.93 | -2.78 (-14.94, 9.44) | 0.69 | 2.40 (-8.99, 13.87) | 0.70 |
| Total effect | 6.44 (-4.09, 18.10) | 0.25 | 6.44 (-4.26, 18.09) | 0.25 | 6.44 (-4.85, 17.75) | 0.25 |
| Proportion mediated% | NA |  | NA |  | NA |  |

Adjusted for age, body mass index and intervention.

Statistically significant associations are shown in bold.

MTF, medial tibiofemoral; LTF, lateral tibiofemoral.

eTable 7. Mediation by osteophytes on the associations between total cartilage defects and changes in knee symptoms in females

| **Outcomes** | **MTF osteophyte**  **β (95% CI)** | ***P*** | **LTF osteophyte**  **β (95% CI)** | ***P*** | **Patellar osteophyte**  **β (95% CI)** | ***P*** |
| --- | --- | --- | --- | --- | --- | --- |
| **Change in total knee pain** |  |  |  |  |  |  |
| Indirect effect | -2.11 (-5.57, 1.04) | 0.19 | 0.26 (-2.53, 3.06) | 0.84 | 1.69 (-0.58, 4.39) | 0.15 |
| Direct effect | **6.36 (1.17, 11.85)** | **0.02** | 4.00 (-1.12, 9.13) | 0.12 | 2.57 (-2.46, 7.44) | 0.30 |
| Total effect | 4.26 (-0.68, 8.80) | 0.08 | 4.26 (-0.47, 8.98) | 0.08 | 4.26 (-0.63, 9.06) | 0.08 |
| Proportion mediated% | NA |  | NA |  | NA |  |
| **Change in weight-bearing pain** |  |  |  |  |  |  |
| Indirect effect | -1.24 (-3.13, 0.68) | 0.21 | 0.40 (-1.22, 2.18) | 0.62 | 1.10 (-0.32, 2.74) | 0.14 |
| Direct effect | 3.12 (-0.26, 6.38) | 0.08 | 1.48 (-2.06, 4.71) | 0.41 | 0.78 (-2.43, 3.96) | 0.62 |
| Total effect | 1.88 (-1.12, 4.68) | 0.23 | 1.88 (-1.21, 4.78) | 0.24 | 1.88 (-1.08, 4.84) | 0.21 |
| Proportion mediated% | NA |  | NA |  | NA |  |
| **Change in non-weight-bearing pain** |  |  |  |  |  |  |
| Indirect effect | -0.87 (-2.42, 0.62) | 0.26 | -0.14 (-1.54, 1.15) | 0.84 | 0.59 (-0.48, 1.80) | 0.29 |
| Direct effect | **3.25 (1.08, 5.80)** | **<0.01** | **2.52 (0.35, 4.79)** | **0.02** | 1.79 (-0.22, 3.78) | 0.08 |
| Total effect | **2.38 (0.47, 4.58)** | **0.02** | **2.38 (0.41, 4.47)** | **0.02** | **2.38 (0.35, 4.38)** | **0.02** |
| Proportion mediated% | NA |  | NA |  | NA |  |
| **Change in stiffness** |  |  |  |  |  |  |
| Indirect effect | -0.28 (-1.66, 1.21) | 0.69 | 0.90 (-0.63, 2.47) | 0.25 | **1.04 (0.05, 2.41)** | **0.04** |
| Direct effect | 0.65 (-1.76, 2.91) | 0.62 | -0.54 (-3.07, 1.84) | 0.66 | -0.68 (-2.78, 1.39) | 0.53 |
| Total effect | 0.37 (-1.48, 2.23) | 0.72 | 0.37 (-1.66, 2.25) | 0.69 | 0.37 (-1.47, 2.30) | 0.66 |
| Proportion mediated% | NA |  | NA |  | NA |  |
| **Change in dysfunction** |  |  |  |  |  |  |
| Indirect effect | 0.27 (-8.82, 10.29) | 0.94 | 4.37 (-6.19, 14.40) | 0.39 | 6.40 (-0.56, 15.13) | 0.08 |
| Direct effect | 7.50 (-6.18, 21.05) | 0.30 | 3.40 (-9.99, 16.38) | 0.62 | 1.37 (-12.30, 14.08) | 0.83 |
| Total effect | 7.77 (-5.64, 20.28) | 0.24 | 7.77 (-5.86, 20.60) | 0.24 | 7.77 (-5.65, 20.71) | 0.25 |
| Proportion mediated% | NA |  | NA |  | NA |  |

Adjusted for age, body mass index and intervention.

Statistically significant associations are shown in bold.

MTF, medial tibiofemoral; LTF, lateral tibiofemoral.

eTable 8. Mediation by osteophytes on the associations between total cartilage volumes and changes in knee symptoms in males

| **Outcomes** | **MTF osteophyte**  **β (95% CI)** | ***P*** | **LTF osteophyte**  **β (95% CI)** | ***P*** | **Patellar osteophyte**  **β (95% CI)** | ***P*** |
| --- | --- | --- | --- | --- | --- | --- |
| **Change in total knee pain** |  |  |  |  |  |  |
| Indirect effect | **-2.78 (-6.72, -0.24)** | **0.03** | **-2.45 (-5.67, -0.29)** | **0.03** | -1.87 (-5.27, 0.83) | 0.18 |
| Direct effect | -1.60 (-12.34, 9.78) | 0.74 | -1.93 (-12.15, 8.35) | 0.74 | -2.52 (-13.11, 8.46) | 0.65 |
| Total effect | -4.38 (-15.47, 6.91) | 0.41 | -4.38 (-14.95, 6.19) | 0.41 | -4.38 (-15.29, 6.49) | 0.43 |
| Proportion mediated% | NA |  | NA |  | NA |  |
| **Change in weight-bearing pain** |  |  |  |  |  |  |
| Indirect effect | **-1.85 (-4.46, -0.12)** | **0.03** | **-1.65 (-3.77, -0.05)** | **<0.05** | -1.21 (-3.70, 0.52) | 0.16 |
| Direct effect | -3.45 (-10.98, 3.86) | 0.36 | -3.65 (-11.36, 3.76) | 0.31 | -4.09 (-11.81, 3.63) | 0.27 |
| Total effect | -5.30 (-13.26, 2.37) | 0.17 | -5.30 (-13.75, 2.15) | 0.18 | -5.30 (-13.19, 2.09) | 0.17 |
| Proportion mediated% | NA |  | NA |  | NA |  |
| **Change in non-weight-bearing pain** |  |  |  |  |  |  |
| Indirect effect | **-0.93 (-2.27, -0.08)** | **0.03** | **-0.81 (-2.03, -0.01)** | **<0.05** | -0.66 (-1.93, 0.24) | 0.16 |
| Direct effect | 1.85 (-2.49, 6.43) | 0.45 | 1.73 (-2.49, 6.90) | 0.45 | 1.58 (-3.04, 6.42) | 0.49 |
| Total effect | 0.92 (-3.37, 5.31) | 0.70 | 0.92 (-3.35, 5.95) | 0.66 | 0.92 (-3.67, 5.63) | 0.69 |
| Proportion mediated% | NA |  | NA |  | NA |  |
| **Change in stiffness** |  |  |  |  |  |  |
| Indirect effect | -0.68 (-1.89, 0.11) | 0.10 | **-1.05 (-2.48, -0.04)** | **0.04** | **-0.57 (-2.05, 0.22)** | **0.20** |
| Direct effect | -0.72 (-5.87, 4.27) | 0.77 | -0.35 (-5.40, 4.71) | 0.82 | -0.84 (-5.83, 4.57) | 0.76 |
| Total effect | -1.41 (-6.67, 3.66) | 0.59 | -1.41 (-6.71, 4.01) | 0.54 | -1.41 (-6.41, 3.91) | 0.60 |
| Proportion mediated% | NA |  | NA |  | NA |  |
| **Change in dysfunction** |  |  |  |  |  |  |
| Indirect effect | **-5.84 (-14.22, -0.13)** | **0.04** | **-6.66 (-15.25, -0.27)** | **0.04** | **-3.58 (-11.81, 1.24)** | **0.19** |
| Direct effect | -20.88 (-49.45, 6.37) | 0.14 | -20.06 (-48.70, 7.94) | 0.16 | -23.14 (-51.51, 5.19) | 0.11 |
| Total effect | -26.72 (-55.06, 1.20) | 0.06 | -26.72 (-55.40, 1.27) | 0.06 | -26.72 (-55.78, 1.46) | 0.06 |
| Proportion mediated% | NA |  | NA |  | NA |  |

Adjusted for age, body mass index and intervention.

Statistically significant associations are shown in bold.

MTF, medial tibiofemoral; LTF, lateral tibiofemoral.

eTable 9. Mediation by osteophytes on the associations between total cartilage volumes and changes in knee symptoms in females

| **Outcomes** | **MTF osteophyte**  **β (95% CI)** | ***P*** | **LTF osteophyte**  **β (95% CI)** | ***P*** | **Patellar osteophyte**  **β (95% CI)** | ***P*** |
| --- | --- | --- | --- | --- | --- | --- |
| **Change in total knee pain** |  |  |  |  |  |  |
| Indirect effect | 1.16 (-3.72, 5.88) | 0.63 | -2.17 (-8.13, 2.93) | 0.36 | -5.16 (-12.44, 0.46) | 0.08 |
| Direct effect | -11.71 (-31.14, 6.94) | 0.22 | -8.38 (-27.30, 10.28) | 0.39 | -5.39 (-23.71, 11.89) | 0.60 |
| Total effect | -10.55 (-29.99, 7.85) | 0.27 | -10.55 (-29.65, 8.28) | 0.26 | -10.55 (-30.78, 7.97) | 0.26 |
| Proportion mediated% | NA |  | NA |  | NA |  |
| **Change in weight-bearing pain** |  |  |  |  |  |  |
| Indirect effect | 0.99 (-1.72, 3.83) | 0.49 | -1.23 (-4.82, 1.92) | 0.42 | -2.89 (-7.41, 0.43) | 0.10 |
| Direct effect | -7.96 (-20.41, 3.52) | 0.18 | -5.74 (-18.47, 5.84) | 0.33 | -4.08 (-16.09, 8.14) | 0.52 |
| Total effect | -6.97 (-19.45, 4.15) | 0.25 | -6.97 (-19.33, 4.77) | 0.24 | -6.97 (-18.81, 5.08) | 0.24 |
| Proportion mediated% | NA |  | NA |  | NA |  |
| **Change in non-weight-bearing pain** |  |  |  |  |  |  |
| Indirect effect | 0.17 (-2.29, 2.39) | 0.90 | -0.94 (-3.97, 1.55) | 0.46 | -2.28 (-5.96, 0.61) | 0.13 |
| Direct effect | -3.75 (-12.52, 4.61) | 0.41 | -2.64 (-11.27, 5.89) | 0.52 | -1.30 (-10.00, 6.80) | 0.71 |
| Total effect | -3.58 (-12.23, 4.68) | 0.41 | -3.58 (-12.44, 4.86) | 0.39 | -3.58 (-12.33, 4.55) | 0.38 |
| Proportion mediated% | NA |  | NA |  | NA |  |
| **Change in stiffness** |  |  |  |  |  |  |
| Indirect effect | 0.32 (-1.71, 2.42) | 0.78 | -1.36 (-4.52, 1.32) | 0.28 | **-2.30 (-5.74, -0.05)** | **0.04** |
| Direct effect | -3.29 (-11.00, 5.18) | 0.44 | -1.62 (-9.67, 6.06) | 0.65 | -0.67 (-8.81, 8.03) | 0.86 |
| Total effect | -2.97 (-10.75, 5.14) | 0.48 | -2.97 (-11.07, 4.92) | 0.44 | -2.97 (-11.08, 5.34) | 0.49 |
| Proportion mediated% | NA |  | NA |  | NA |  |
| **Change in dysfunction** |  |  |  |  |  |  |
| Indirect effect | -2.85 (-20.63, 10.42) | 0.65 | -9.64 (-33.66, 8.13) | 0.28 | -16.76 (-40.09, 0.22) | 0.05 |
| Direct effect | -18.42 (-72.72, 36.98) | 0.56 | -11.63 (-63.44, 42.18) | 0.67 | -4.51 (-65.00, 52.19) | 0.86 |
| Total effect | -21.27 (-77.62, 34.06) | 0.46 | -21.27 (-77.40, 34.40) | 0.47 | -21.27 (-83.30, 32.37) | 0.44 |
| Proportion mediated% | NA |  | NA |  | NA |  |

Adjusted for age, BMI and intervention.

Statistically significant associations are shown in bold.

MTF, medial tibiofemoral; LTF, lateral tibiofemoral.

eTable 10. Mediation by follow-up osteophytes on the associations between baseline total cartilage defects and changes in knee symptoms.

| **Outcomes** | **MTF osteophyte**  **β (95% CI)** | ***P*** | **LTF osteophyte**  **β (95% CI)** | ***P*** | **Patellar osteophyte**  **β (95% CI)** | ***P*** |
| --- | --- | --- | --- | --- | --- | --- |
| **Change in total knee pain** |  |  |  |  |  |  |
| Indirect effect | 0.66 (-1.03, 2.38) | 0.43 | 1.47 (-0.25, 3.33) | 0.08 | **1.04 (0.08, 2.15)** | **0.03** |
| Direct effect | 2.87 (-0.02, 5.44) | 0.05 | 2.06 (-1.18, 5.77) | 0.18 | 2.49 (-0.32, 5.33) | 0.08 |
| Total effect | **3.53 (0.50, 6.58)** | **0.02** | **3.53 (0.54, 6.36)** | **0.02** | **3.53 (0.58, 6.56)** | **0.02** |
| Proportion mediated% | NA |  | NA |  | **29%** |  |
| **Change in weight-bearing pain** |  |  |  |  |  |  |
| Indirect effect | 0.32 (-0.77, 1.40) | 0.55 | 0.99 (-0.10, 2.19) | 0.08 | **0.57 (0.01, 1.30)** | **0.05** |
| Direct effect | 1.73 (-0.31, 4.08) | 0.10 | 1.06 (-1.19, 3.44) | 0.32 | 1.48 (-0.31, 3.58) | 0.10 |
| Total effect | **2.05 (0.09, 4.16)** | **0.04** | **2.05 (0.13, 4.10)** | **0.04** | **2.05 (0.11, 3.96)** | **0.04** |
| Proportion mediated% | NA |  | NA |  | **28%** |  |
| **Change in non-weight-bearing pain** |  |  |  |  |  |  |
| Indirect effect | 0.34 (-0.53, 1.13) | 0.40 | 0.48 (-0.28, 1.31) | 0.23 | **0.47 (0.02, 0.99)** | **0.04** |
| Direct effect | 1.17 (-0.23, 2.74) | 0.11 | 1.03 (-0.40, 2.59) | 0.17 | 1.04 (-0.15, 2.38) | 0.08 |
| Total effect | **1.51 (0.24, 2.88)** | **0.02** | **1.51 (0.23, 2.80)** | **0.02** | **1.51 (0.34, 2.77)** | **0.01** |
| Proportion mediated% | NA |  | NA |  | **31%** |  |
| **Change in stiffness** |  |  |  |  |  |  |
| Indirect effect | 0.25 (-0.56, 1.06) | 0.51 | **0.88 (0.06, 1.73)** | **0.04** | **0.64 (0.19, 1.17)** | **<0.01** |
| Direct effect | 0.27 (-0.94, 1.98) | 0.51 | -0.36 (-1.68, 1.53) | 0.88 | 0.12 (-1.21, 1.53) | 0.86 |
| Total effect | 0.52 (-0.83, 1.91) | 0.45 | 0.52 (-0.82, 1.89) | 0.46 | 0.52 (-0.87, 1.90) | 0.50 |
| Proportion mediated% | NA |  | NA |  | NA |  |
| **Change in dysfunction** |  |  |  |  |  |  |
| Indirect effect | 3.99 (-0.98, 9.21) | 0.12 | **6.82 (2.36, 12.80)** | **<0.01** | **3.33 (0.89, 6.79)** | **0.01** |
| Direct effect | 2.97 (-5.15, 10.67) | 0.40 | 0.14 (-9.33, 9.28) | 0.99 | 3.63 (-3.70, 12.07) | 0.30 |
| Total effect | 6.96 (-1.77, 15.70) | 0.11 | 6.96 (-0.75, 16.10) | 0.09 | 6.96 (-1.20, 15.66) | 0.10 |
| Proportion mediated% | NA |  | NA |  | NA |  |

Adjusted for age, sex, body mass index and vitamin D supplement.

Statistically significant associations are shown in bold.

MTF, medial tibiofemoral; LTF, lateral tibiofemoral.

eTable 11. Mediation by follow-up osteophytes on the associations between baseline total cartilage volume and changes in knee symptoms.

| **Outcomes** | **MTF osteophyte**  **β (95% CI)** | ***P*** | **LTF osteophyte**  **β (95% CI)** | ***P*** | **Patellar osteophyte**  **β (95% CI)** | ***P*** |
| --- | --- | --- | --- | --- | --- | --- |
| **Change in total knee pain** |  |  |  |  |  |  |
| Indirect effect | -1.44 (-3.69, 0.39) | 0.12 | **-2.21 (-4.59, -0.48)** | **<0.01** | **-2.12 (-4.69, -0.32)** | **0.01** |
| Direct effect | -4.86 (-15.01, 3.55) | 0.21 | -4.09 (-13.44, 4.15) | 0.28 | -4.18 (-14.45, 4.59) | 0.28 |
| Total effect | -6.30 (-15.43, 2.89) | 0.16 | -6.30 (-16.21, 3.11) | 0.19 | -6.30 (-15.90, 2.69) | 0.17 |
| Proportion mediated% | NA |  | NA |  | NA |  |
| **Change in weight-bearing pain** |  |  |  |  |  |  |
| Indirect effect | -0.72 (-2.12, 0.37) | 0.19 | **-1.33 (-2.72, -0.17)** | **0.02** | **-1.12 (-2.73, -0.03)** | **0.04** |
| Direct effect | -5.77 (-12.39, 0.66) | 0.08 | -5.16 (-11.50, 1.37) | 0.12 | -5.37 (-11.81, 1.39) | 0.12 |
| Total effect | **-6.49 (-13.45, -0.21)** | **0.04** | **-6.49 (-13.44, -0.30)** | **0.04** | **-6.49 (-13.20, -0.29)** | **0.04** |
| Proportion mediated% | NA |  | **20%** |  | **17%** |  |
| **Change in non-weight-bearing pain** |  |  |  |  |  |  |
| Indirect effect | -0.72 (-1.73, 0.12) | 0.10 | **-0.88 (-1.93, -0.09)** | **0.03** | **-1.00 (-2.25, -0.18)** | **<0.01** |
| Direct effect | -0.52 (-4.26, 3.83) | 0.89 | -0.36 (-4.15, 3.77) | 0.92 | -0.24 (-4.15, 3.77) | 0.97 |
| Total effect | -1.24 (-5.43, 2.89) | 0.59 | -1.24 (-5.62, 3.04) | 0.53 | -1.24 (-5.41, 2.82) | 0.54 |
| Proportion mediated% | NA |  | NA |  | NA |  |
| **Change in stiffness** |  |  |  |  |  |  |
| Indirect effect | -0.34 (-1.28, 0.44) | 0.40 | **-0.89 (-2.03, -0.08)** | **0.03** | **-1.06 (-2.34, -0.23)** | **<0.01** |
| Direct effect | -2.60 (-6.82, 1.88) | 0.26 | -2.05 (-6.18, 2.07) | 0.37 | -1.88 (-5.99, 2.52) | 0.42 |
| Total effect | -2.94 (-7.22, 1.51) | 0.19 | -2.94 (-7.58, 1.47) | 0.19 | -2.94 (-7.23, 1.36) | 0.19 |
| Proportion mediated% | NA |  | NA |  | NA |  |
| **Change in dysfunction** |  |  |  |  |  |  |
| Indirect effect | -4.74 (-12.21, 0.28) | 0.07 | **-8.09 (-16.30, -2.12)** | **<0.01** | **-5.91 (-13.14, -0.81)** | **<0.01** |
| Direct effect | **-27.55 (-53.03, -2.91)** | **0.03** | -24.20 (-48.90, 0.52) | 0.06 | **-26.38 (-53.00, -0.66)** | **0.04** |
| Total effect | **-32.29 (-59.72, -6.32)** | **0.02** | **-32.29 (-58.36, -5.73)** | **0.02** | **-32.29 (-58.13, -5.62)** | **0.02** |
| Proportion mediated% | NA |  | **25%** |  | **18%** |  |

Adjusted for age, sex, body mass index and vitamin D supplement.

Statistically significant associations are shown in bold.

MTF, medial tibiofemoral; LTF, lateral tibiofemoral
